# *p*-Cymene Protects Mice Against Lipopolysaccharide-Induced Acute Lung Injury by Inhibiting Inflammatory Cell Activation

**DOI:** 10.3390/molecules17078159

**Published:** 2012-07-06

**Authors:** Guanghong Xie, Na Chen, Lanan Wassy Soromou, Fang Liu, Ying Xiong, Qianchao Wu, Hongyu Li, Haihua Feng, Guowen Liu

**Affiliations:** College of Animal Science and Veterinary Medicine, Jilin University, Changchun 130062, China

**Keywords:** *p*-cymene, lipopolysaccharide, acute lung injury

## Abstract

The objective of this study was to test the hypothesis that *p*-cymene can attenuate acute lung injury induced by lipopolysaccharide (LPS) *in vivo*. In the mouse model of LPS-induced acute lung injury, intraperitoneal preconditioning with *p*-cymene resulted in a significant reduction of pro-inflammatory cytokines (TNF-α, IL-1β and IL-6), lung water gain, inﬂammatory cell inﬁltration, lung tissue myeloperoxidase activity. In addition, *p*-cymene blocked the phosphorylation of IκBα protein and mitogen-activated protein kinases (MAPK) signaling pathway activation. Histopathologic examination of lung tissue indicated that *p*-cymene treatment markedly decreased focal thickening, congestion, pulmonary edema, and inflammatory cells infiltration. The results showed that *p*-cymene had a protective effect on LPS-induced ALI in mice.

## 1. Introduction

**Figure 1:**
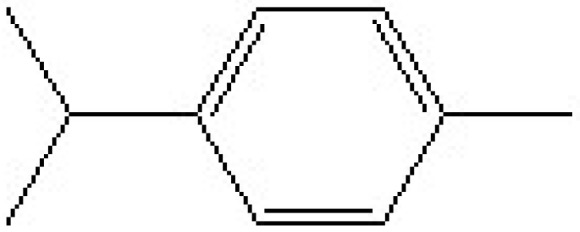
The chemical structure of *p*-cymene.

*p*-Cymene (4-isopropyltoluene or 1-isopropyl-4-methylbenzene, Figure 1), an important chemical widely used for syntheses of *p*-cresol [1], is a natural aromatic hydrocarbon which occurs in the oils of many gymnospermic and angiospermic plants [2,3]. *p*-Cymene is also an important intermediate used in pharmaceutical industries and for the production of fungicides, pesticides, as flavoring agent [4]. It is one of the main constituents of the essential oil from species of *Protium* [5]. Several studies have shown that species rich in *p*-cymene show antinociceptive activity in rodents [6,7,8,9]. Rattanachaikunsopon *et al.* [10], have reported that *p*-cymene has been known to exhibit antimicrobial activity against a variety of Gram-positive and negative bacteria. *p*-Cymene has also been shown to be an effective agent in the management of orofacial pain [11], but so far, the anti-inflammatory effects of *p*-cymene on acute lung injury (ALI) has not yet been studied.

Acute lung injury (ALI) is a complex syndrome marked by increased vascular permeability resulting in tissue edema and profound hypoxia [12]. ALI is a neutrophil-mediated, widespread inﬂammatory condition affecting the pulmonary parenchyma. It is a syndrome that affects around one in 10 general intensive care unit patients. Mortality of patients with ALI is between 22% and 58% depending on severity [13]. In an animal model of lung injury, direct exposure of LPS to lung tissue triggers ALI which is characterized by increased capillary permeability, interstitial and alveolar edema and an influx of circulating inflammatory cells [14].

Downstream signal transduction pathways that have been shown to participate in mediating lung inflammatory responses include the NF-κB, MAPK, and phosphotidylinositol 3-kinase pathways. NF-κB has been shown to be upregulated in animal models as well as in humans with ALI [15]. The basic injury of ALI is the destruction of the pulmonary capillary endothelium and alveolar epithelium by polymorphonuclear neutrophils (PMNs) and activated macrophages which constitute a source of numerous inflammatory mediators, including tumor necrosis factor (TNF)-α, interleukins (IL-1β and 6), and arachidonic acid (AA) and oxygen metabolites [16,17]. These mediators of inflammation play a fundamental role in the degradation of alveolar matrix that is associated with a variety of pulmonary diseases [18,19]. The development of the new therapeutic agents capable of inducing anti-inflammatory responses concurrent with minimal systemic side effects is crucial for treating inflammation. Therefore, the present study was undertaken to examine the effect of *p*-cymene on ALI induced by intranasal instillation of LPS in BALB/c mice and to investigate its possible mechanisms of action.

## 2. Results and Discussion

### 2.1. Effect of p-Cymene on Inflammatory Cell Count in BALF of LPS-Induced Mice

**Figure 2:**
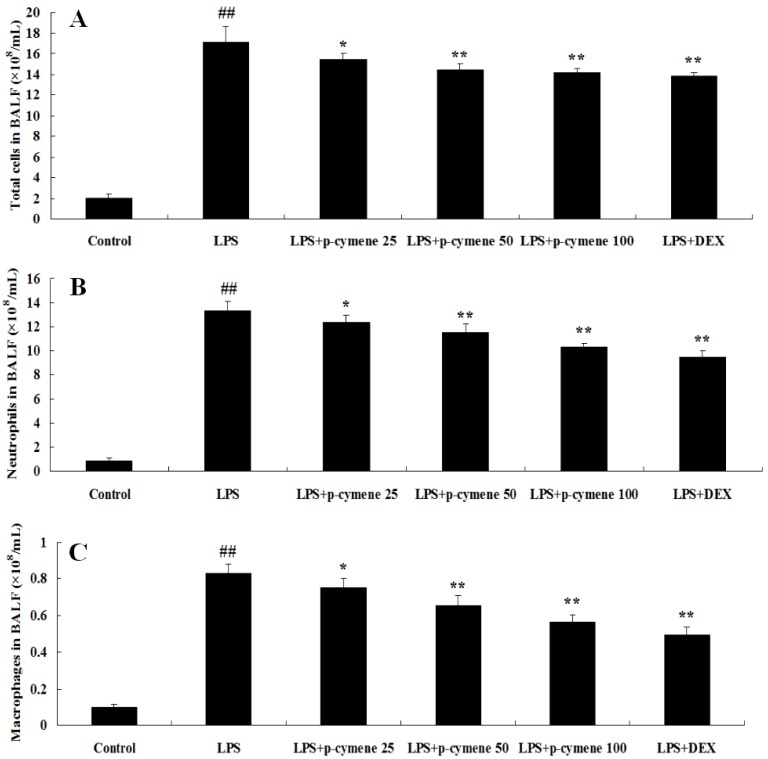
Effects of *p*-cymene on the number of total cells, neutrophils, and macrophages in the BALF of mice with LPS-induced ALI. Mice were given an intraperitoneal injection of *p*-cymene (25, 50 or 100 mg/kg) 1 h prior to an i.n. administration of LPS. BALF was collected at 7 h following LPS challenge to measure the number of total cells (**A**), neutrophils (**B**), and macrophages (**C**). The values presented are the mean ± SEM (n = 6 in each group). *^##^ p* < 0.01 *vs.* control group, * p < 0.05, ** p < 0.01 *vs.* LPS group.

BALF was collected at 7 h after LPS administration and the number of total cells, neutrophils and macrophages was analyzed. As shown in Figure 2, the number of total cells, neutrophils and macrophages increased significantly after LPS challenge, compared to the control group (*p* < 0.01). Furthermore, pretreatment with *p*-cymene (25, 50 or 100 mg/kg) or DEX (5 mg/kg) was found to significantly decrease the number of total cells (*p* < 0.01; Figure 2A), neutrophils (*p* < 0.01; Figure 2B) and macrophages (*p* < 0.01; Figure 2C).

### 2.2. Effects of p-Cymene on the Lung W/D Ratio and BALF Protein Concentration in LPS-Induced ALI Mice

**Figure 3:**
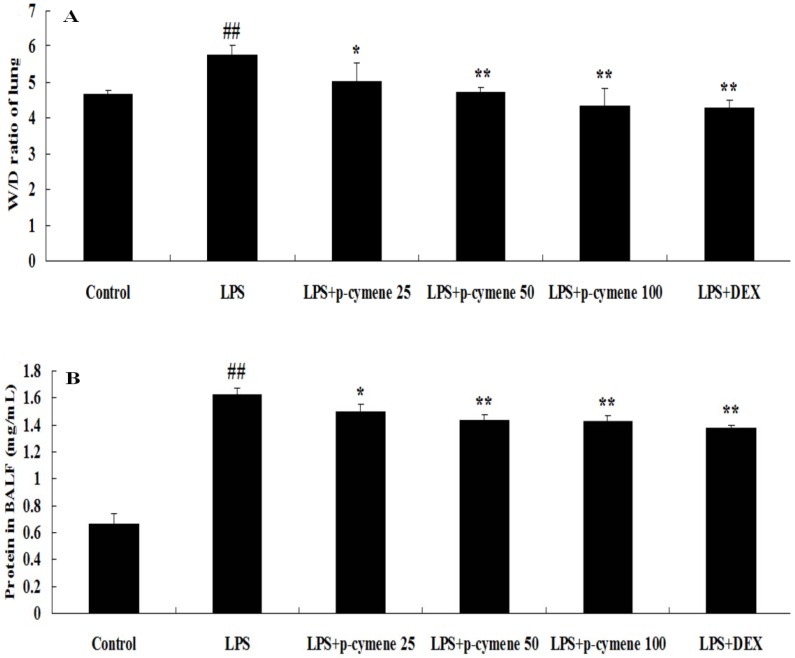
Effects of *p*-cymene on the lung W/D ratio and total protein concentration in the BALF of mice with LPS-induced ALI. Mice were given an intraperitoneal injection of *p*-cymene (25, 50 or 100 mg/kg) 1 h prior to an i.n. administration of LPS. The lung W/D ratio (**A**) and total protein concentration in the BALF (**B**) were determined at 7 h after LPS challenge. The values presented are the mean ± SEM (n = 6 in each group). *^##^ p* < 0.01 *vs.* control group, * p < 0.05, ** p < 0.01 *vs.* LPS group.

BALF was collected at 7 h after LPS administration. As shown, LPS challenge resulted in a significant increase in the lung W/D ratio (*p* < 0.01; Figure 3A) and total protein concentration (*p* < 0.01; Figure 3B). However, pretreatment with *p*-cymene (25, 50 or 100 mg/kg) and DEX (5 mg/kg) significantly decreased the lung W/D ratio and the total protein concentration (*p* < 0.01 *vs.* control group, *p* < 0.05, *p* < 0.01 *vs.* LPS group).

### 2.3. Effect of p-Cymene on LPS-Induced Release of Cytokines in the BALF of Mice

**Figure 4:**
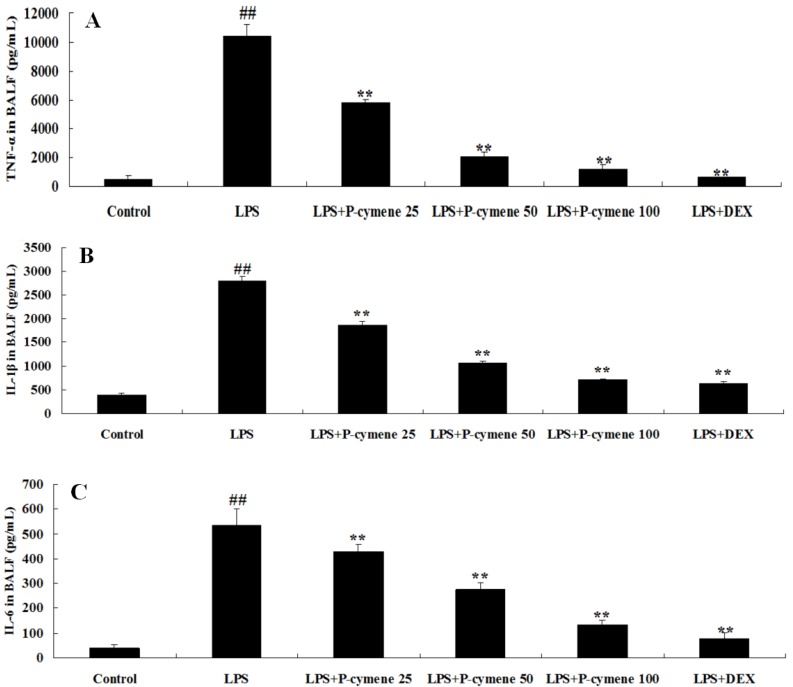
Effects of *p*-cymene on the production of inﬂammatory cytokine TNF-α, IL-1β and IL-6 in the BALF of mice with LPS-induced ALI. Mice were given an intraperitoneal injection of *p*-cymene (25, 50 or 100 mg/kg) 1 h prior to administration of LPS. BALF was collected at 7 h following LPS challenge to analyze the inﬂammatory cytokines TNF-α (**A**), IL-1β (**B**) and IL-6 (**C**). The values presented are the mean ± SEM (n = 6 in each group). *^##^ p* < 0.01 *vs.* control group, * p < 0.05, ** p < 0.01 *vs.* LPS group.

BALF was collected at 7 h after LPS administration and the levels of cytokines in BALF were measured by ELISA. As shown in Figure 4, TNF-α, IL-1β and IL-6 levels in the BALF of LPS-treated mice were significantly increased compared to those in control group. *p*-Cymene and DEX significantly reduced the secretion of TNF-α (*p* < 0.01; Figure 4A), IL-1β (*p* < 0.01; Figure 4B) and IL-6 (*p* < 0.01; Figure 4C).

### 2.4. Effect of p-Cymene on MPO Activity

**Figure 5:**
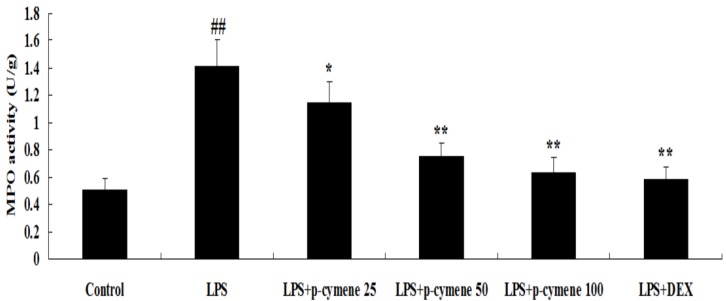
Effects of *p*-cymene on MPO activity in lungs of LPS-instilled mice. Seven hours after LPS instillation, lung homogenates were prepared for determination of MPO activity. Data are presented as the mean ± SEM (n=6 in each group). *^##^ p* < 0.01 *vs.* control group, * p < 0.05, ** p < 0.01 *vs.* LPS group.

The MPO activity was determined to assess neutrophil accumulation within pulmonary tissues. As shown in Figure 5, LPS challenge resulted in significant increases in lung MPO activity compared with the control group (*p* < 0.01). Pretreatment with *p*-cymene (especially at a dose of 50 and 100 mg/kg) and DEX apparently reduced this increase, (*p* < 0.01 *vs.* LPS group). 

### 2.5. Effect of p-Cymene on Histopathological Changes in the Lung Tissue of LPS-induced ALI Mice

**Figure 6:**
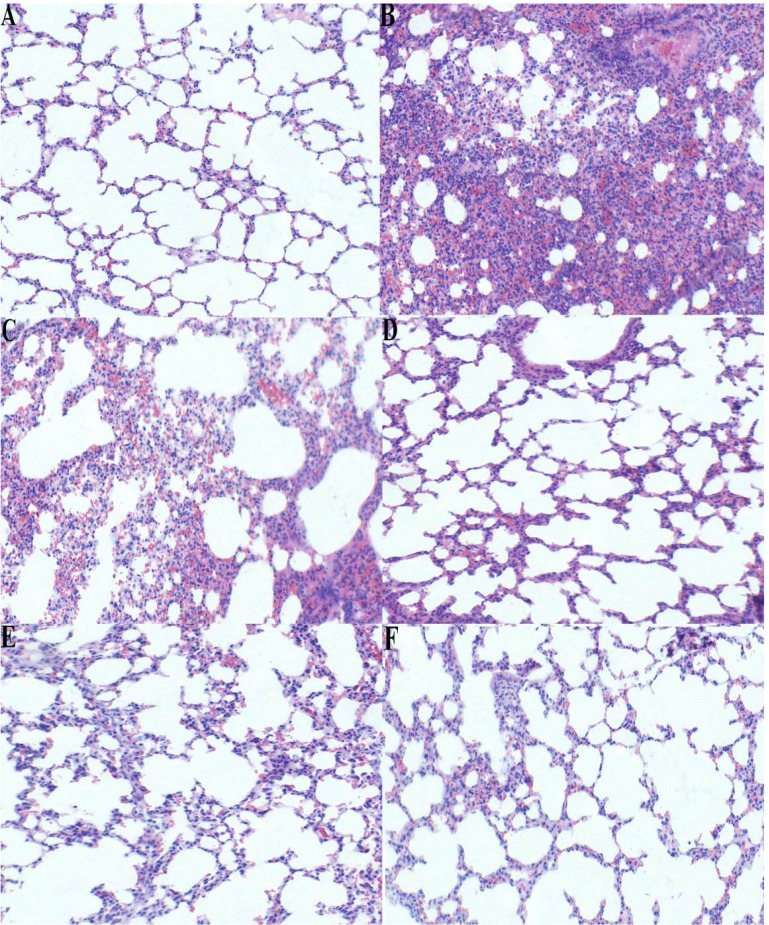
Effect of *p*-cymene on histopathological changes in lung tissues in LPS-induced ALI mice (×100). Mice were given a intraperitoneal injection of *p*-cymene (25, 50, 100 mg/kg) 1 h prior to an i.n. administration of LPS. Lungs (n = 3) from each experimental group were processed for histological evaluation at 7 h after LPS challenge. Lungs were prepared for H&E staining.Control group(**A**): microphotograph showing normal structure of the lung. LPS group(**B**): microphotograph showing histopathological changes (focal thickening, congestion) of the lung. *p*-Cymene group(**C**, **D**, **E**) and DEX group(**F**): microphotograph showing decreased histopathological changes of the lung. A, B, C, D, E and F magnification 100×.

To evaluate the histological changes after *p*-cymene treatment in LPS-induced mice, lung sections were subjected to hematoxylin and eosin staining. Normal pulmonary histology with intact structures and clear pulmonary alveoli in control group is shown in Figure 6A. In the LPS group (Figure 6B), lung tissue were significantly damaged and showed signs of characteristic histological changes, including pulmonary congestion and oedema, infiltration of the tissue and alveoli with inflammatory cells, thickening of the alveolar wall and alveolar collapse. However, *p*-cymene (25, 50 or 100 mg/kg) and DEX (5 mg/kg) ameliorated these histopathological changes (Figure 6C–F).

### 2.6. Effect of p-Cymene on LPS-Induced MAPK and NF-κB Pathways

**Figure 7:**
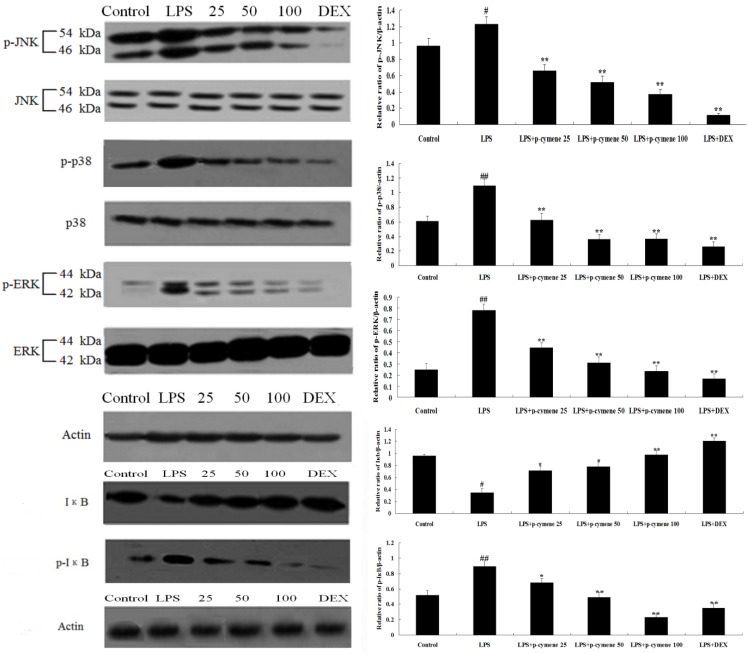
Effect of *p*-cymene on LPS-induced MAPK and NF-κB pathways. To induce ALI, LPS was instilled intranasally 1 h after intraperitoneal administration of *p*-cymene (25, 50 or 100 mg/kg). Quantification of protein expression was normalized to β-actin using a densitometer (Imaging System). The data are representative of three independent experimentsand expressed as mean ± SEM. *p* < 0.01 indicates significant differences from the unstimulated control group. *^##^ p* < 0.01 *vs.* control group, * p < 0.05, ** p < 0.01 *vs.* LPS group.

To investigate the mechanism by which *p*-cymene inhibits LPS-induced production of inflammatory cytokines, the MAPK pathways (ERK1/2, p38 and JNK phosphorylation) were measured by western blot analysis. As shown in Figure 7, LPS stimulation significantly increased the phosphorylation of JNK, ERK1/2 and p38 MAPK. In contrast, *p*-cymene (25, 50 or 100 mg/kg) and DEX (5 mg/kg) inhibited the phosphor-JNK, phosphor-ERK and phosphor-p38. 

Additionally, we evaluated the effect of *p*-cymene on LPS-induced NF-κB activation to confirm whether the inhibitory effects of *p*-cymene on cytokine production were regulated by the NF-κB signaling pathway. As shown in Figure 7, LPS-induced IκBα degradation was blocked by pretreatment with *p*-cymene. To determine whether this IκBα degradation was related to IκBα phosphorylation, the effect of *p*-cymene on LPS-induced p-IκBα was examined. It was found that *p*-cymene significantly inhibited LPS-induced IκBα phosphorylation in a dose-dependent manner (*p* < 0.05, *p* < 0.01).

### 2.7. Discussion

During endotoxemia, severe lung inflammation causes ALI, an important clinical problem with significant mortality. Lung inflammation is characterized by increased pulmonary inflammatory cell sequestration and the production of pro-inflammatory mediators, which leads to the development of protein leakage in alveolar space, reduced lung compliance, and finally impaired lung function [20]. In this investigation, various effects of *p*-cymene and its possible mechanisms in LPS-induced acute lung injury were investigated: histopathological changes, proinflammatory cytokines in BALF, lung edema formation, inflammatory cell infiltration, myeloperoxidase (MPO) activity, and NF-κB and MAPK signaling pathways were determined.

Lung is one of the most frequently involved organs in a variety of complications in the immunocompromised host and ALI induced by LPS is the major cause of death [21]. Polymorphonuclear leukocytes (PMNs) have been shown to be responsible for ALI and edema in animal models of ALI, and PMNs oxidants and proteases injure cells of the alveolar–capillary membrane [22]. They play an important role in mediating ALI. In the normal lower respiratory tract, macrophages represent the majority of phagocytes whereas PMNs are almost absent. However, PMNs can accumulate within the lung structure, just as in the aftermath of injury, trauma or infection [23]. MPO is a major constituent of neutrophil cytoplasmic granules. The total activity of MPO in a tissue is therefore a direct measure of neutrophil sequestration in that tissue [24]. In this study, we found that MPO activity in lungs was dramatically increased after LPS administration and pretreatment of *p*-cymene significantly lowered the increased MPO activity. The pulmonary endothelium participates in the exchange of water and solutes between the blood and the interstitium. It was found that *p*-cymene attenuated the development of pulmonary edema which was increased by LPS administration. Under normal conditions, a small amount of fluid is filtered across the endothelial monolayer and drained by the lymph. Fluid ﬁltration is limited by the continuous nature of the endothelium, with multiple connections between the cells called tight and adherens junctions [25]. In our study, pre-administration of *p*-cymene decreased the number of total cells, PMNs, and macrophages; more importantly, *p*-cymene was confirmed to reduce LPS-stimulated pulmonary morphological changes (congestion, septal thickening, and edema). In our experiment, DEX was used as positive drug and our results also confirmed its protective effects suggesting that DEX attenuated LPS-induced inflammatory responses in ALI.

During the investigation, it was essential to assess the effect of *p*-cymene on LPS-induced production of inﬂammatory cytokines which induce the activation of various cells [26]. TNF-α is produced earlier than most other cytokines. Although the major sources of TNF-α are thought to bePMNs and alveolar macrophages, recent data show that TNF-α is also produced and secreted by alveolar type II pneumocytes. IL-1β activates neutrophils and induces up-regulation of adhesion molecules on both leukocytes and endothelium [27]. After 7 h pretreatment with *p*-cymene, the lung tissue concentration of TNF-α, IL-1β and IL-6 in the *p*-cymene groups was significantly lower than that in the LPS group (Figure 4, *p* < 0.05). These results are in agreement with our earlier findings that demonstrated the inhibitory effect of *p*-cymene against proinflammatory cytokine production in RAW 264.7 cells *in vitro* (unpublished data). 

Inhibiting NF-κB and MAPK activities in alveolar macrophages may contribute to the *p*-cymene’s inhibitory effect on cytokine production. NF-κB pathway is known to play a key role in the pathogenesis of ALI. Stimulated with LPS, NF-κB is activated by phosphorylation, enters the nucleus and regulates the expression of inﬂammatory cytokines. Therefore, inhibiting NF-κB activation is crucial for treating inﬂammation [28,29]. The MAPKs are a group of signaling molecules that play a critical role in the regulation of cell growth and differentiation, as well as in the control of cellular responses to cytokines and stresses [30,31]. Once activated by dual phosphorylation on tyrosine and threonine, the MAPKs modulate the functional responses of cells through phosphorylation of transcription factors and activation of other kinases. The three major MAPK proteins, p38 MAPK, JNK and ERK, are thought to play different roles in inﬂammatory diseases in different capacities [32]. Among these MAPK groups, JNK and ERK are known to play important roles as upstream regulators of the induced expression of inflammatory mediators in response to cytokines, stress, and cytoskeletal reorganization. They are both activated after *in vivo* LPS exposure in models of acute lung injury [33]. Therefore, inhibition of JNK, ERK or p38 activity has potential as an effective therapeutic strategy in interventions of inflammatory cascade-associated lung injury. Recent studies have shown that pharmacological inhibitors of NF-κB and MAP kinases strongly affect the production of inflammatory mediators [34,35]. In this study, NF-κB and MAPK activities were activated in LPS-induced lung injury. In contrast LPS-induced NF-κB, ERK1/2, JNK and p38/MAPK activation in lung tissue was inhibited by *p*-cymene pretreatment. This study suggests that *p*-Cymene may be a promising agent for the prevention of ALI. 

## 3. Experimental

### 3.1. Chemicals and Reagents

*p*-Cymene (Mol. Weight: 134.22, Det. Purity: 99.5%) was purchased from Dr. Ehrenstorfer (Augsburg, Germany); dexamethasone (DEX) was purchased from Changle Pharmaceutical Co. (Xinxiang, Henan, China). LPS (*E. coli* 055: B5) and dimethylsulfoxide (DMSO) were purchased from Sigma Chemical Co. (St. Louis, MO, USA). Mouse TNF-α, IL-1β and IL-6 enzyme-linked immunosorbent assay (ELISA) kits were purchased from Biolegend (San Diego, CA, USA). The myeloperoxidase (MPO) determination kit was purchased from Jiancheng Bioengineering Institute of Nanjing (Nanjing, Jiangsu Province, China). Mouse monoclonal phosphospecific p42-p44 ERK antibodies, mouse monoclonal phosphospecific p46-p54 JNK antibodies, mouse monoclonal phosphospecific p38 antibodies, mouse mAb Phospho-IκBα and rabbit mAb IκBα were purchased from Cell Signaling Technology Inc. (Beverly, MA, USA). HRP-conjugated goat-mouse antibodies and goat anti-rabbit antibodies were purchased from GE Healthcare (Buckinghamshire, UK). All other chemicals were of reagent grade.

### 3.2. Animals

Pathogen-free BALB/c male mice, weighing approximately 18 to 20 g, were purchased from the Center of Experimental Animals of Baiqiuen Medical College of Jilin University (Jilin, China). The mice were fed a standard diet and water *ad libitum* and housed in microisolator cages under standard conditions (temperature: 24 ± 1 °C, relative humidity: 40%–80%). Before experimentation, the mice were housed for 2–3 days to adapt them to their environment. All animal experiments were performed in accordance with the guide for the Care and Use of Laboratory Animals published by the US National Institutes of Health.

### 3.3. LPS-induced ALI Model

Mice were randomly assigned into the following six groups: Control, LPS, LPS + *p*-cymene and LPS + Dexamethasone (LPS + DEX). LPS + DEX group served as positive control. *p*-cymene (25, 50 or 100 mg/kg) and DEX (5 mg/kg) were given intraperitoneally. Mice from control and LPS groups received an equal volume of phosphate-buffered saline (PBS) instead of *p*-cymene or DEX. One hour later, mice were anesthetized with diethyl ether and 10 μg of LPS was instilled intranasally in 50 μL PBS to induce acute lung injury; control mice were given 50 μL PBS without LPS. Collection of bronchoalveolar lavage fluid (BALF) was performed three times through a tracheal cannula with 0.5 mL of autoclaved PBS, instilled up to a total volume of 1.3 mL. The mice were then killed by diethyl ether asphyxiation, and their lungs were placed into a sterile centrifuge tube and stored at −80 °C.

### 3.4. Inflammatory Cell Counts of BALF

BALF samples were centrifuged (3,000 rpm, 4 °C, 10 min) to pellet the cells. The cell pellets were resuspended in PBS to obtain total cell counts using a haemocytometer. Cytospins were prepared for differential cell counts by staining with the Wright-Giemsa staining method.

### 3.5. Lung Wet-to-Dry Weight (W/D) Ratio and Protein Analysis

After the mice were killed by diethyl ether asphyxiation, the lungs were excised, blotted dry, weighed to obtain the “wet” weight, and placed in an oven at 80 °C for 48 h to obtain the “dry” weight. The ratio of wet lung to dry lung was calculated to assess tissue oedema. Protein concentration in the supernatant of BALF was quantified using the bicinchoninic acid (BCA) method to evaluate vascular permeability in the airways.

### 3.6. Cytokine Assays

The concentrations of TNF-α, IL-1β and IL-6 in the supernatants of the BALF were measured by enzyme-linked immunosorbent assay (ELISA) kits in accordance with the manufacturer’s instructions.

### 3.7. Pulmonary Myeloperoxidase Activity in ALI Mice

MPO activity, which reflects the parenchymal infiltration of neutrophils and macrophages, was measured as described previously [36,37]. Mice were killed 7 h after LPS administration under diethyl ether anaesthesia. The right lung was excised and 100 mg was homogenized in 50 mM hydroxyethyl- piperazine ethanesulfonic acid (HEPES) pH 8.0 containing 0.5% cetyltrimethylammonium bromide (CTAB) and subjected to three freeze–thaw cycles. The homogenate was centrifuged at 13,000 ×g for 30 min at 4 °C, and the cell-free extracts were stored at −20 °C until further use. The MPO activity was assayed using a mouse MPO ELISA kit. Samples were diluted in phosphate citrate buffer pH 5.0.

### 3.8. Histopathological Evaluation of the Lung Tissue

Histopathological examination was performed on mice that were not subject to BALF collection. Lungs were fixed with 10% neutral formalin, dehydrated with graded alcohol dilutions and embedded in paraffin, and sliced subsequently. After staining with haematoxylin and eosin (H&E), pathological changes in the lung tissues were observed under a light microscope.

### 3.9. Western Blot Analysis

Seven hours after LPS injection, lung tissues were harvested and frozen immediately in liquid nitrogen for storage until homogenisation. Samples were homogenised on ice using cell lysis buffer for Western and IP (Beyotime Institute of Biotechnology, Haimen, China) with the addition of a 1:100 dilution of phenylmethyl sulfonyl fluoride (PMSF). The homogenate was centrifuged at 14,000 ×g for 10 min at 4 °C and the protein concentrations were determined using Bio-Rad protein assay reagent (Bio-Rad, Munich, Germany). The supernatant was aliquoted and stored at −80 °C after removing a small aliquot for protein quantification. Aliquots of the lysates were separated by 12% SDS-polyacrylamide gel and transferred to a polyvinylidene fluoride (PVDF) membrane (Bio-Rad) with a glycine transfer buffer [192 mM glycine, 25 mM Tris-HCl (pH 8.8), 20% methanol (v/v)]. After the blots were blocked for 2 h with 5% (w/v) non-fat dry milk, the membrane was incubated overnight with specific primary antibody at 4 °C and washed in Tween 20/Tris-buffered saline (TTBS). The membrane was incubated with a horseradish peroxidase-conjugated secondary antibody for 1 h at room temperature and washed with TTBS again. Then, the immunoreactive proteins were detected by using an enhanced chemiluminescence (ECL) Plus Western Blotting Detection System (Amersham Life Science, South Clearbrook, MN, USA).

### 3.10. Statistical Analysis

All values are expressed as mean ± SEM. Comparison between groups was assessed with one-way analysis of variance (ANOVA; Dunnett’s *t*-test) and Student’s t-test. Statistical difference was accepted at *p* < 0.05 or *p* < 0.01.

## 4. Conclusions

In summary, this study demonstrates that pretreatment with *p*-cymene totally prevented LPS-induced increased production of TNF-α, IL-1β and IL-6. Furthermore, *p*-cymene can attenuate lung inflammatory responses in mouse model of acute lung injury through NF-κB and MAPK inactivation.

## References

[1] Zhang Q.G., Bi L.W., Zhao Z.D., Chen Y.P., Li D.M., Gu Y., Wang J., Chen Y.X., Bo C.Y., Liu X.Z. (2010). Application of ultrasonic spraying in preparation of *p*-cymene by industrial dipentene dehydrogenation. Chem. Eng. J..

[2] Benchaar C., Calsamiglia S., Chaves A.V., Fraser G.R., Colombatto D., McAllister T.A., Beauchemin K.A. (2008). A review of plant-derived essential oils in ruminant nutrition and production. Anim. Feed Sci. Technol..

[3] Singh H.P., Kohli R.K., Batish D.R., Kaushal P.S. (1999). Allelopathy of gymnospermous trees. J. Forest. Res..

[4] Selvaraj M., Pandurangan A., Seshadri K.S., Sinha P.K., Krishnasamy V., Lal K.B.  (2002). Comparison of mesorporous A1-MCM-41 molecular sieves in the production of *p*-cymene for isopropylation of toluene. J. Mol. Catal. A-Chem..

[5] Siani A.C., Garrido I.S., Carvalho E.S., Ramos M.F.S. (1999). Evaluation of anti-inflammatory-related activity of essential oils from the leaves and resin of species of *Protium*. J. Ethnopharmacol..

[6] Oliveira F.A., Costa C.L.S., Chaves M.H., Almeida F.R.C., Cavalcante I.J.M., Lima A.F., Lima-Júnior R.C.P., Silva R.M., Campos A.R., Santos F.A. (2005). Attenuation of capsaicin-induced acute and visceral nociceptive pain by alpha- and beta-amyrin, a triterpene mixture isolated from *Protium heptaphyllum* resin in mice. Life Sci..

[7] Otuki M.F., Lima F.V., Malheiros A., Filho V.C., Monache F.D.M., Yunes R.A., Calixto J. (2001). Evaluation of the antinociceptive action caused by ether fraction and a triterpene isolated from resin of *Protium kleinii*. Life Sci..

[8] Bispo M.D., Mourão R.H.V., Franzotti E.M., Bomfim K.B.R., Arrigoni-Blank M.F., Moreno M.P.N., Marchioro M., Antoniolli A.R. (2001). Antinociceptive and antiedematogenic effects of the aqueous extract of *Hyptis pectinata* leaves in experimental animals. J. Etnopharmacol..

[9] Ramezani M., Hosseinzadeh H., Samizadeh S. (2004). Antinociceptive effects of *Zataria multiflora* Boiss fractions in mice. J. Ethonopharmacol..

[10] Rattanachaikunsopon P., Phumkhachorn P. (2010). Synergistic antimicrobial effect of nisin and *p*-cymene on *Salmonella enterica* serovar Typhi *in vitro* and on ready-to-eat food. Biotechnol. Biochem..

[11] Santana M.F., Quintans-Júnior L.J., Sócrates Cavalcanti C.H., Makson Oliveira G.B., Guimarães A.G., Cunha E.S., Melo M.S., Márcio Santos R.V., Adriano Araújo A.S., Bonjardim L.R. (2011). *p*-Cymene reduces orofacial nociceptive response in mice. Rev. Bras. Farmacogn..

[12] Peng X.Q., Damarla M., Skirball J., Nonas S., Wang X.Y., Han E.J., Hasan E.J., Cao X., Boueiz A., Damico R. (2010). Protective role of PI3-kinase/Akt/eNOS signaling in mechanical stress through inhibition of p38 mitogen-activated protein kinase in mouse lung. Acta Pharmacol. Sin..

[13] The Acute Respiratory Distress Syndrome Network (2000). Ventilation with lower tidal volumes as compared with traditional tidal volumes for acute lung injury and the acute respiratory distress syndrome. N. Engl. J. Med..

[14] Zhang X., Huang H., Yang T., Ye Y., Shan J., Yin Z., Luo L. (2010). Chlorogenic acid protects mice against lipopolysaccharide-induced acute lung injury. Injury.

[15] Severgnini M., Takahashi S., Rozo L.M., Homer R.J., Kuhn C., Jhung J.W., Perides G., Steer M., Hassoun P.M., Fanburg B.L. (2004). Activation of the STATpathway in acute lung injury. Am. J. Physiol. Lung Cell Mol. Physiol..

[16] Windsor A.C., Mullen P.G., Fowler A.A. (1993). Acute lung injury: What have we learned from animal models?. Am. J. Med. Sci..

[17] Sutcliffe A.J. (1994). The future of ARDS. Injury.

[18] Stockley R.A. (1994). The role of proteinases in the pathogenesis of chronic bronchitis. Am. J. Respir. Crit. Care Med..

[19] Venaille T.J., Ryan G., Robinson B.W. (1998). Epithelial cell damage is induced by neutrophil-derived, not pseudomonas-derived, proteases in cystic fibrosis sputum. Respir. Med..

[20] Chu P.Y., Chien S.P., Hsu D.Z., Liu M.Y. (2010). Protective effect of sesamol on the pulmonary inflammatory response and lung injury in endotoxemic rats. Food Chem. Toxicol..

[21] Zhang X., Huang H., Yang T., Ye Y., Shan J., Yin Z., Luo L. (2010). Chlorogenic acid protects mice against lipopolysaccharide-induced acute lung injury. Injury.

[22] Brigham K.L. (1982). Mechanisms of lung injury. Clin. Chest Med..

[23] Sibille Y., Reynolds H.Y. (1990). Macrophages and polymorphonuclear neutrophils in lung defence and injury. Am. Rev. Respir. Dis..

[24] Bradley P.P., Priebat D.A., Christensen R.D., Rothestein G. (1982). Measurement of cutaneous inflammation: Estimation of neutrophil content with an enzyme marker. J. Invest. Dermatol..

[25] Maniatis N.A., Kotanidou A., Catravas J.D., Orfanos S.E. (2008). Pathomechanisms in acute lung injury. Vascul. Pharmacol..

[26] Goodman R.B., Strieter R.M., Martin D.P., Steinberg K.P., Milberg J.A., Maunder R.J., Kunkel S.L., Walz A., Hudson L.D., Martin T.R. (1996). Inflammatory cytokines in patients with persistence of the acute respiratory distress syndrome. Am. J. Respir. Crit. Care Med..

[27] Zhu Y.B., Wang Q., Liu Y.L., Li X.F., Li J.A., Lü X.D., Ling F., Liu A.J., Fan X.M. (2010). Effect of partial liquid ventilation on oleic acid-induced inflammatory responses in piglets. Chin. Med. J..

[28] Abraham E. (2000). NF-kappaB activation. Crit. Care Med..

[29] Blackwell T.S., Christman J.W. (1997). The role of nuclear factor-kappa B in cytokine gene regulation. Am. J. Respir. Cell Mol. Biol..

[30] Molnar A., Theodoras A.M., Zon L.I., Kyriakis J.M. (1997). Cdc42Hs, but not Rac 1, inhibits serum-stimulated cell cycle progressin at G1/S through a mechanism requiring p38/RK. J. Biol. Chem..

[31] Takenaka K., Mcriguchi T., Nishida E. (1998). Activation of the protein kinase p38 in the spindle assembly checkpoint and mitotic arrest. Science.

[32] Zhang L., Li H.Z., Gong X., Luo F.L., Wang B., Hu N., Wang C.D., Zhang Z., Wan J.Y. (2010). Protective effects of Asiaticoside on acute liver injury induced by lipopolysaccharide/D-galactosamine in mice. Phytomedicine.

[33] Lee H.S., Kim H.J., Moon C.S., Chong Y.H., Kang J.L. (2004). Inhibition of c-Jun NH_2_-terminal kinase or extracellular signal-regulated kinase improves lung injury. Respir. Res..

[34] Van den Blink B., Juffermans N.P., ten Hove T., Schultz M.J., van Deventer S.J., van der Poll T., Peppelenbosch M.P. (2001). p38 Mitogen-activated protein kinase inhibition increases cytokine release by macrophages *in vitro* and during infection *in vivo*. J. Immunol..

[35] Scherle P.A., Jones E.A., Favata M.F., Daulerio A.J., Covington M.B., Nurnberg S.A., Magolda R.L., Trzaskos J.M. (1998). Inhibition of MAP kinase prevents cytokine and prostaglandin E2 production in lipopolysaccharide-stimulated monocytes. J. Immunol..

[36] Ysebaert D.K., de Greef K.E., Vercauteren S.R., Ghielli M., Verpooten G.A., Eyskens E.J. (2000). Identification and kinetics of leukocytes after severe ischaemia/reperfusion renal injury. Nephrol. Dial. Transplant..

[37] Peng X., Hassoun P.M., Sammani S., McVerry B.J., Burne M.J., Rabb H. (2004). Protective effects of sphingosine1-phosphate in murine endotoxin-induced inflammatory lung injury. Am. J. Respir. Crit. Care Med..

